# Isoalantolactone Attenuates LPS-Induced Acute Lung Injury, an Effect Associated with Suppressed MAPK/STAT3 Activation and Epithelial-Mesenchymal Transition

**DOI:** 10.7150/ijms.126267

**Published:** 2026-08-12

**Authors:** Chia-Yang Li, Chung-Wei Liu, Hsin-Han Tseng, Yu-Hsin Tseng, Shu-Chi Wang, Po-Len Liu, Hsin-En Wu, Yuan-Ru Chen, Sheng-I Lee, Tzu-Chieh Lin, Ching-Tang Chang, Xin-Yi Lin, I-Chen Chen

**Affiliations:** 1 Graduate Institute of Medicine, College of Medicine, Kaohsiung Medical University, Kaohsiung 80708, Taiwan.; 2 Center for Disease Multi-Omics Research, Kaohsiung Medical University, Kaohsiung 80708, Taiwan.; 3 Department of Medical Research, Kaohsiung Medical University Hospital, Kaohsiung 80756, Taiwan.; 4 Department of Respiratory Therapy, College of Medicine, Kaohsiung Medical University, Kaohsiung 80708, Taiwan.; 5 Department of Pediatrics, Kaohsiung Medical University Hospital, Kaohsiung 80756, Taiwan.; 6 Department of Medical Laboratory Science and Biotechnology, Kaohsiung Medical University, Kaohsiung 80708, Taiwan.; 7 Graduate Institute of Clinical Medicine, College of Medicine, Kaohsiung Medical University, Kaohsiung 80708, Taiwan.; 8 Division of Cardiology, Department of Internal Medicine, Kaohsiung Medical University, Kaohsiung 80708, Taiwan.; 9 Department of Pediatrics, School of Medicine, College of Medicine, Kaohsiung Medical University, Kaohsiung 80708, Taiwan.; 10 Department of Pediatrics, Kaohsiung Medical University Gangshan Hospital, Kaohsiung 820111, Taiwan.

**Keywords:** acute lung injury, epithelial-mesenchymal transition, isoalantolactone, interleukin-6, MAPK signaling pathway

## Abstract

**Background:**

Acute lung injury (ALI) is characterized by a high mortality rates and acute inflammation that compromises the epithelial and endothelial barriers of the respiratory system. Isoalantolactone (IsoA) is a natural compound derived from* Inula helenium* that has been shown to exhibit anti-inflammatory activity. However, its ability to preserve epithelial barrier integrity or modulate mitogen-activated protein kinase/signal transducer and activator of transcription 3 (MAPK/STAT3) signaling and epithelial-mesenchymal transition (EMT) has not been evaluated in ALI.

**Methods:**

Human bronchial epithelial cells (HBEC3-KT and BEAS-2B) were stimulated with lipopolysaccharide (LPS) with or without IsoA to assess cytotoxicity and cytokine release. In vivo, female C57BL/6 mice were pretreated with IsoA (10-20 mg/kg, intraperitoneally) or vehicle 1 h before LPS challenge (1 mg/kg, intratracheally). At 4 h, lung tissues and bronchoalveolar lavage fluid (BALF) were collected for histopathology and to measure total protein, cellular composition, and cytokine levels (tumor necrosis factor-α [TNF-α], interleukin-6 [IL-6], monocyte chemoattractant protein-1 [MCP-1]); leukocyte subsets were quantified by multicolor flow cytometry. Lung homogenates were analyzed using Western blot for phosphorylated/total extracellular signal-regulated kinase 1/2 (ERK1/2), c-Jun N-terminal kinase 1/2 (JNK1/2), p38 MAPKs, STAT3, and EMT markers (E-cadherin, N-cadherin, snail).

**Results:**

In LPS-stimulated HBEC3-KT and BEAS-2B cells, IsoA significantly reduced IL-6 secretion. In the murine model of LPS-induced ALI, IsoA administration alleviated lung tissue damage, decreased inflammatory cell infiltration, and reduced IL-6 and TNF-α concentration in BALF and serum. Mechanistically, IsoA attenuated the phosphorylation of ERK, JNK, and p38 MAPKs and suppressed STAT3 activation in lung tissues. Furthermore, IsoA attenuated EMT, as evidenced by decreased N-cadherin and snail expressions and restored E-cadherin expression.

**Conclusions:**

IsoA alleviates LPS-induced ALI by reducing MAPK/STAT3 activation and EMT-associated epithelial injury. These findings suggest that IsoA may be a promising candidate for the targeted modulation of inflammatory lung injury, and that further preclinical and translational studies are justified.

## Introduction

Acute lung injury (ALI) and its more severe manifestation, acute respiratory distress syndrome (ARDS), are life-threatening syndromes associated with substantial mortality 1-3. They are characterized by disruption of the alveolar epithelial barrier and dysregulated inflammatory signaling 3-5, which facilitates the influx of inflammatory cells into the lung parenchyma and amplifies the host response. This exaggerated response is orchestrated by a network of pro-inflammatory cytokines that are central to disease pathogenesis, including interleukin-1β (IL-1β), tumor necrosis factor-α (TNF-α), IL-6, IL-8, and epithelial-derived monocyte chemoattractant protein-1 (MCP-1, also known as CCL2), which mediates critical macrophage crosstalk and impairs barrier repair 6-8. Notably, IL-6 has been shown to drive the phosphorylation of signal transducer and activator of transcription 3 (STAT3), thereby promoting neutrophil recruitment and influencing antibacterial defenses 9-13. Additional pathways implicated in lung inflammation include Janus kinase (JAK)/STAT3, nuclear factor-κB (NF-κB), and mitogen-activated protein kinase (MAPK) signaling 11, 13-17.

The three major pathways involving the MAPK family are extracellular signal-regulated kinases (ERKs), c-Jun N-terminal kinases (JNKs), and p38 MAPKs. The MAPK pathway has been shown to be activated by lipopolysaccharide (LPS) 17, and to be integral to the production of IL-6 and MCP-1, further escalating inflammation 18. At the same time, neutrophils and macrophages infiltrate the alveolar space, contributing to hypoxemia and tissue injury.

Epithelial-mesenchymal transition (EMT), a process defined by the loss of epithelial characteristics and the acquisition of mesenchymal traits, is increasingly recognized as a critical event in the pathogenesis of ALI 19, 20. Although extensively studied in cancer, the contribution of EMT to epithelial injury in ALI and its therapeutic modulation remain less well defined, and it would thus be interesting to investigate whether targeting EMT could favorably influence epithelial integrity during ALI.

Despite advances in lung-protective ventilation, effective pharmacologic therapies for ALI/ARDS remain elusive 7, 21. Isoalantolactone (IsoA) is a natural compound derived from *Inula helenium* that has been shown to exhibit anti-allergic, antioxidant, and anti-cancer activities, as well as anti-inflammatory effects in preclinical models 22-29. For example, He et al. reported that IsoA reduced the production of nitric oxide, prostaglandins, and cytokines such as IL-6 and TNF-α in LPS-induced sepsis models 30. In addition, prior studies have implicated nuclear factor erythroid 2-related factor 2 (Nrf2) signaling and tumor necrosis factor receptor-associated factor 6 (TRAF6) ubiquitination in IsoA-mediated protection in lung injury, suggesting that additional mechanisms may involve 31, 32. However, the pharmacologic targets of IsoA have yet to be fully elucidated 22. Therefore, the aim of this study was to evaluate the protective effects of IsoA against LPS-induced ALI, with a specific focus on MAPK modules, the IL-6/STAT3 axis, and EMT.

## Methods

### Mice

Female C57BL/6 mice (6-8 weeks old, weighing 17-19 g) were obtained from the National Laboratory Animal Center (Taipei, Taiwan). Female mice were used to maintain consistency with our previous studies and minimize variability 12.

All experimental procedures were approved by the Committee on the Ethics of Animal Experiments at Kaohsiung Medical University (Permit Number: 111199). Mice were housed in a temperature-controlled room (25 ± 1°C) on a 12-h light/dark cycle with ad libitum access to food and water.

### Cell culture

Human bronchial epithelial cells (HBEC3-KT and BEAS-2B) were purchased from the American Type Culture Collection (Catalog Nos. CRL-4051 and CRL-3588, Manassas, VA, USA). The cells were cultured in Ham's F-12K and RPMI 1640 media supplemented with 10% fetal bovine serum and 1% penicillin-streptomycin (Corning, Corning, NY, USA) and incubated in a humidified atmosphere containing 5% CO₂ at 37°C.

### Cell viability assay

The MTT assay was performed on HBEC3-KT and BEAS-2B cells to assess the cytotoxic effects of IsoA (Catalog No. CFN98107, ChemFaces, Wuhan, Hubei, China). Cells were seeded at a density of 5 × 10⁴ cells/mL in a 96-well plate. After overnight adherence, the cells were pretreated with 0-10 μM IsoA (dissolved in 0.5% dimethyl sulfoxide to prepare a stock solution, with serial dilutions made using phosphate-buffered saline [PBS]) and subsequently treated with LPS (1 μg/mL, derived from Escherichia coli O111:B4, Sigma Aldrich, St. Louis, MO, USA) for 24 h. The final concentration of DMSO was maintained at less than 0.1% to avoid potential cytotoxicity.

Next, 10 μL of MTT solution (5 mg/mL in PBS, Sigma Aldrich, St. Louis, MO, USA) was added to each well and thoroughly mixed. After 4 hours of incubation, the medium was aspirated, and the resulting formazan crystals were dissolved by adding 100 μL of acidified isopropanol (0.04 N HCl). The absorbance of the solubilized formazan (purple color) was measured using a microplate reader at 570 nm.

For the verification of EMT-associated markers at an extended time point, BEAS-2B cells were seeded in 6-well plates at a density of 2.5 × 10⁵ cells per well and allowed to adhere overnight. The cells were pretreated with IsoA (5 or 10 μM) or vehicle for 1 h, followed by stimulation with LPS (1μg/mL) for 24 h. Subsequently, total cellular proteins were extracted for the determination of vimentin expression via Western blot analysis.

### ALI model

The mice were randomly assigned to four groups: control, LPS (1 mg/kg), IsoA 10 mg/kg + LPS, and IsoA 20 mg/kg + LPS. IsoA was administered intraperitoneally (i.p.) 1 h before intratracheal (i.t.) LPS instillation. The controls received PBS in lieu of IsoA and LPS. All mice were euthanized 4 h after PBS or LPS administration, and bronchoalveolar lavage fluid (BALF) and lung tissues were collected for analysis. This 4-h observational endpoint was selected based on our previously established ALI model 12, as it is the optimal time window for capturing the peak activation of early inflammatory signaling cascades, particularly the MAPKs and STAT3 pathways.

### Enzyme-linked immunosorbent assay (ELISA)

TNF-α, MCP-1, and IL-6 concentrations in cell culture supernatants, murine BALF, and serum were measured using ELISA kits (Origene Technologies, Rockville, MD, USA) according to the manufacturer's protocols.

### Multicolor flow cytometry

The staining panel for immune cell subsets in BALF was developed based on previously established protocols 12, 33. Briefly, BALF cells were stained at 4°C for 30 min using the following fluorochrome-conjugated monoclonal antibodies: FITC-conjugated anti-Ly6G (1A8; BD Biosciences, San Jose, CA, USA), PE-conjugated anti-Siglec-F (E50-2440; BD Biosciences), APC-conjugated anti-B220 (RA3-6B2; BD Biosciences), APC-conjugated anti-CD3 (145-2C11; BD Biosciences), PerCP/Cy5.5-conjugated anti-CD11b (M1/70; BioLegend, San Diego, CA, USA), and eFluor 450-conjugated anti-CD11c (N41B; Invitrogen, Eugene, OR, USA). Cell viability was assessed using Live/Dead Fixable Red dye (Invitrogen). To ensure data accuracy, single-stained compensation beads and cells were used to calculate and apply color compensation matrix, correcting for spectral overlap among the fluorophores. Gating strategies were established using unstained cells and fluorescence minus one (FMO) to account for background fluorescence and ensure population specificity. After staining and washing, samples were analyzed by multiparameter flow cytometry using an LSR II instrument (BD Biosciences), and the data were analyzed with FlowJo software (version 10; Tree Star, Ashland, OR, USA). The gating strategy used to identify immune cell populations in BALF is shown in Supplementary Figure S2.

### Western blot analysis

Lung tissues were homogenized in RIPA buffer supplemented with protease and phosphatase inhibitors (Sigma Aldrich, St. Louis, MO, USA) and centrifuged at 12,000 × g for 10 min. The supernatants were collected, and total protein concentrations were determined using a BCA protein assay kit (Thermo Scientific, Waltham, MA, USA). β-tubulin was used as an internal control, and all target protein concentrations were normalized to their respective loading controls. Protein expressions were analyzed using specific primary antibodies, including phospho-ERK1/2 (CST#4370, Cell Signaling Technology, Danvers, MA, USA), ERK1/2 (CST#4695, Cell Signaling Technology), phospho-JNK1/2 (CST#9255, Cell Signaling Technology), JNK1/2 (CST#9258, Cell Signaling Technology), phospho-p38 MAPK (CST#4511, Cell Signaling Technology), p38 MAPK (CST#8690, Cell Signaling Technology), phospho-STAT3 (CST#9145, Cell Signaling Technology), STAT3 (CST#9139, Cell Signaling Technology), N-cadherin (CST#4061, Cell Signaling Technology), E-cadherin (BD#610182, BD Biosciences), snail (CST#3879, Cell Signaling Technology), vimentin (CST#5741, Cell Signaling Technology, Danvers, MA, USA), β-actin (C4, Santa Cruz Biotechnology, Santa Cruz, CA, USA), and β-tubulin (GTX629630, GeneTex, Irvine, CA, USA). The membranes were washed three times with Tris-PBS containing 0.05% Tween-20 and incubated with horseradish peroxidase (HRP)-conjugated secondary antibodies (1:5,000; Santa Cruz Biotechnology) for 1 h at room temperature. The membranes were then washed three times with PBS containing 0.1% Tween-20 (PBST) for 10 min each, followed by a final wash in PBS to remove residual Tween-20. Protein signals were detected using enhanced chemiluminescence (Thermo Scientific) and visualized with a Bio-Rad ChemiDoc XRS+ imaging system (Bio-Rad Laboratories, Inc., Hercules, CA, USA) following an additional wash.

### Histopathological assessment with hematoxylin and eosin (H&E) staining

Lung tissues were fixed in 4% paraformaldehyde at a constant pressure to ensure standardized inflation for histology. They were embedded in paraffin, sectioned at a thickness of 4 µm, and stained with H&E. Pathological changes in the lung tissues were examined under an optical microscope. Histological scoring parameters included edema, alveolar and interstitial inflammation, alveolar and interstitial hemorrhage, atelectasis, and hyaline membrane formation. Each parameter was scored on a scale of 0 to 4 as follows: no injury (0), injury in 25% of the field (1), injury in 50% of the field (2), injury in 75% of the field (3), and injury throughout the field (4). The maximum score was 28 16, 34.

### Statistical analysis

A p-value <0.05 was considered statistically significant. Sample sizes were determined by power analysis to achieve a power of 0.80 at a significance level of 0.05.

A minimum of six samples per group were calculated to be required for 95% power to detect a difference with a significance (α) of 0.05, using the two-sided two-sample t test. Data are expressed as mean ± SEM. The normality of data distribution was assessed using the Shapiro-Wilk test, and homogeneity of variances was evaluated using the Brown-Forsythe test. For data meeting these assumptions, differences between groups were analyzed using one-way analysis of variance (ANOVA) followed by Tukey's post hoc test for multiple comparisons. Non-parametric variables were compared using the Mann-Whitney rank-sum test. All statistical analyses were conducted using GraphPad Prism 10 software (GraphPad Software Inc., San Diego, CA, USA). The significance for all statistical tests is shown in the figures.

## Results

### IsoA was non-cytotoxic and suppressed LPS-induced cytokines/chemokines in airway epithelial cells

We first assessed the impact of IsoA on cell viability using the MTT assay. Cells were pre-treated with varying concentrations of IsoA (2.5-10 µM) for 1 h prior to LPS exposure (1 µg/ml) for 24 h. As shown in Figure 1A, no cytotoxic effects were observed at IsoA concentrations of up to 10 µM in either the HBEC3-KT cells.

We then measured concentrations of IL-6, a major pro-inflammatory cytokine in ALI 35. The results showed that IsoA significantly reduced IL-6 production in LPS-stimulated HBEC3-KT cells in a concentration-dependent manner (Figures 1B). We further analyzed the production of MCP-1, a critical chemokine that regulates the migration and infiltration of monocytes/macrophages 36. Similar to IL-6, IsoA treatment significantly decreased MCP-1 production in the LPS-stimulated HBEC3-KT cells, also in a concentration-dependent manner (Figures 1C). Consistent results were observed in BEAS2B cells, where IsoA exhibited no cytotoxicity (Figure 1D) and similarly decreased IL-6 (Figure 1E) and MCP-1 (Figure 1F) production.

Analysis of other pro-inflammatory cytokines including TNF-α, IL-1β, and IL-12, as well as nitric oxide production, revealed no detectable contents in the supernatants from LPS-stimulated HBEC3-KT and BEAS2B cells (Figure S1).

### IsoA attenuated the production of inflammatory cytokines in LPS-induced ALI mice

To evaluate the effects of IsoA on inflammatory cytokine production in LPS-induced ALI mice, the concentrations of TNF-α and IL-6 were quantified in BALF and serum using ELISA. Following LPS administration, there were significant increases in the concentrations of IL-6 and TNF-α in BALF compared to the PBS-treated control group (Figures 2A and 2B). Notably, the administration of IsoA at concentrations of 10 mg/kg and 20 mg/kg markedly reduced the concentrations of TNF-α and IL-6 in BALF (Figures 2A and 2B). In addition, the serum concentration of IL-6 was significantly elevated in the LPS-treated group compared to the PBS-treated controls, but significantly reduced following treatment with IsoA at a concentration of 20 mg/kg (Figure 2C). These findings indicated that IsoA could effectively suppress the production of the pro-inflammatory cytokines IL-6 and TNF-α in BALF and IL-6 in serum in the LPS-induced ALI mice.

### IsoA reduced the infiltration of inflammatory cells in the lungs of LPS-induced ALI mice

We further examined the effect of IsoA on the infiltration of inflammatory cells in LPS-induced ALI mice. LPS administration significantly enhanced granulocyte and lymphocyte recruitment in the lungs of the mice (Figures 3A and 3B). Treatment with IsoA at a dose 10 mg/kg or 20 mg/kg notably decreased the density of granulocytes in BALF compared to the LPS-stimulated group; however, there was no significant difference in lymphocyte density between the LPS-stimulated and IsoA-treated groups (Figure 3B). In addition, the density of macrophages did not differ significantly among the LPS-stimulated, control, and IsoA-treated groups. These findings suggested that IsoA could effectively reduce the infiltration of granulocytes in the lungs of mice subjected to LPS-induced ALI.

### IsoA suppressed the phosphorylation of MAPKs and toll-like receptor 4 (TLR4) in lung tissues of LPS-induced ALI mice

As both MAPKs and TLR4 are critical components of the immune response to pathogens and play significant roles in the regulation of inflammation 37, we next investigated the effect of IsoA on the phosphorylation of MAPKs and expression of TLR4 in lung tissues of LPS-induced ALI mice. The results showed that the intratracheal administration of LPS markedly enhanced the phosphorylation of MAPKs including p38 and p-JNK, and the expression of TLR4 (Figures 4A-4E). In contrast, IsoA (10 and 20 mg/kg) treatment significantly decreased the phosphorylation of p38, p-JNK, and p-ERK (Figures 4B-4D). Moreover, the expression of TLR4 was notably reduced in the groups treated with 10 mg/kg and 20 mg/kg of IsoA compared to the LPS-stimulated group (Figure 4E).

### IsoA modulated STAT3 and EMT activity in lung tissues

Because IL-6 is a key driver of STAT3 phosphorylation and EMT has been implicated in epithelial injury, we analyzed STAT3 and EMT markers using Western blot. The results showed that intratracheal LPS administration significantly increased the expressions of, N-cadherin and snail, and decreased the expression of E-cadherin (Figures 5A-5E). Treatment with IsoA at doses of 10 mg/kg and 20 mg/kg significantly decreased the ratio of total STAT3 to β-tubulin (Figure 5B). In addition, N-cadherin expression was reduced at a dose of 20 mg/kg IsoA but not at 10 mg/kg IsoA compared to the LPS-treated group (Figure 5C). In contrast, E-cadherin expression was increased at doses of both 10 mg/kg and 20 mg/kg IsoA compared to the LPS group (Figure 5D). Finally, the expression of snail was decreased at a dose of 20 mg/kg IsoA but remained unchanged at a dose of 10 mg/kg IsoA compared to the LPS-stimulated group (Figure 5E).

To further validate the inhibitory effect of IsoA on EMT at the cellular level, we examined the expression of vimentin in LPS-stimulated BEAS-2B cells *in vitro*. Consistent with the tissue findings, Western blot analysis revealed that treatment with IsoA (10 μM) significantly attenuated the expression of vimentin (Figures 5F and 5G). These results suggested that IsoA could effectively modulate STAT3 and EMT signaling in both lung tissues and bronchial epithelial cells in response to LPS-induced lung injury.

### IsoA decreased lung injury in LPS-induced ALI mice

H&E staining was performed to elucidate the impact of IsoA on histological alterations in the LPS-induced ALI mice. The results showed that LPS administration led to substantial infiltration of inflammatory cells around the alveoli (Figure 6A). In contrast, treatment with IsoA markedly reduced this infiltration. Furthermore, the ALI severity score for quantifying histological damage was significantly higher in the LPS-treated group compared to the controls (Figure 6B). However, treatment with 20 mg/kg IsoA significantly reduced the ALI score (Figure 6B). These findings indicated that IsoA could effectively attenuate both inflammatory cell infiltration and histological damage in the lung tissues of LPS-induced ALI mice.

## Discussion

IsoA is a bioactive compound known for its pleiotropic anti-inflammatory and antioxidant activities 22. While the roles of MAPKs, STAT3, and EMT in ALI have been previously described, our study is the first to demonstrate that IsoA acts as a multi-target modulator specifically in the bronchial epithelium to provide integrated protection. We found that IsoA not only reduced classical inflammatory cell infiltration and cytokine storms (IL-6, TNF-α) but also preserved epithelial integrity, such as suppressing the STAT3-driven EMT, a dual mechanism that distinguishes IsoA from conventional single-target anti-inflammatory agents.

The influx of inflammatory cells and the release of inflammatory mediators are the hallmarks of ALI 7, 38, 39. Accordingly, we observed prominent granulocytic recruitment after LPS challenge, which was attenuated by IsoA, along with reductions in acute inflammatory factors in BALF. Beyond myeloid-driven inflammation, current research increasingly emphasizes the role of the bronchial epithelium in cytokine production 40, 41. Our *in vitro* results showed that IsoA directly suppressed IL-6 release from LPS-stimulated BEAS-2B cells, supporting that a direct epithelial protective mechanism contributed to the reductions in inflammatory cytokines in the alveolar space. These findings indicate that IsoA moderates the early neutrophil-dominated response that contributes to alveolar injury and edema formation.

In line with the role of the bronchial epithelium in cytokine production 40, 41, our results showed that IsoA suppressed IL-6 release from LPS-stimulated bronchial epithelial cells, supporting a direct epithelial mechanism. Unlike previous studies that focused on myeloid-driven inflammation, our results in BEAS-2B cells and lung tissues showed that IsoA significantly mitigated the mesenchymal transition (downregulation of N-cadherin, snail, and vimentin; upregulation of E-cadherin). This is particularly relevant because EMT is now recognized not just as a precursor to fibrosis, but as a driver of acute barrier dysfunction 20, 42. By maintaining E-cadherin contents, IsoA likely preserves the tightness of the alveolar-capillary barrier, thereby reducing the edema and neutrophil influx observed in our histological data.

It has been demonstrated that the mesenchymal transition can be attenuated by regulating key signaling pathways such as NF-κB and STAT3, which are crucial in the initiation and progression of this process 20, 43, 44. In our study, the concurrent suppression of STAT3 protein levels and EMT markers by IsoA aligns with the established role of STAT3 as a potent driver of EMT in the pulmonary epithelium. While our observations at 4 hours post-LPS capture the early molecular priming of the epithelium rather than a full phenotypic transition, this integrated regulation underscores the potential of IsoA to preserve the alveolar barrier by preventing the breakdown of epithelial junctions during acute inflammation.

It is important to note that while our results demonstrated that IsoA effectively reduced the activation of MAPKs (p38 and JNK) and STAT3, these effects may be indirect. Previous mechanistic studies have identified upstream targets of IsoA that could explain our observations. For example, IsoA has been reported to activate the Nrf2/HO-1 signaling pathway 31, which exerts potent anti-inflammatory effects and can negatively regulate MAPK activation. In addition, another study suggested that IsoA inhibits the ubiquitination of TRAF6, a critical scaffold protein in the TLR4 signaling cascade, thereby preventing the downstream phosphorylation of MAPKs and NF-κB 32. Our findings of reduced TLR4 expression and suppressed signaling are consistent with these reports, suggesting that IsoA likely acts on a more proximal node of the inflammatory cascade, with MAPK/STAT3 suppression being a significant downstream consequence 23. Further studies, such as molecular docking or surface plasmon resonance (SPR), are needed to identify the precise primary binding target of IsoA in lung epithelial cells.

Interestingly, we observed that the induction of mature IL-1β was less pronounced in the BEAS-2B cells. The production of mature IL-1β is a highly regulated two-step process involving the NLRP3 inflammasome. This relatively attenuated induction may be due to the inherent physiological characteristics of bronchial epithelial cells, which may have a higher threshold for inflammasome activation compared to myeloid cells. This suggests that the protective effects of IsoA in this model may primarily target the NF-κB-dependent priming stage or structural remodeling pathways (EMT), rather than the complete NLRP3-mediated conversion process within the observed sampling window.

Despite the significant findings, this study has several limitations. First, the in vivo experiments were conducted at a single 4-h time point, which captures early inflammatory signaling rather than the full peak of ALI pathophysiology typically seen at 24-48 h. Second, IsoA was administered prophylactically prior to LPS induction. While this preventive strategy effectively demonstrates the protective potential of IsoA against imminent inflammatory injury, it does not fully reflect the clinical reality where ALI/ARDS patients typically require therapeutic interventions after disease onset. Third, while our power analysis supported the sample size (n = 6), this relatively small cohort may limit the detection of more subtle biological effects. Fourth, only female adult mice were used in this study to ensure experimental consistency and minimize biological variability, as previously described in similar LPS-induced lung injury models 12, 45. However, this may limit the generalizability of our results, as sex-dependent differences in immune responses and drug metabolism are well-documented in adult rodents. Furthermore, the lack of significant IL-1β and IL-12 induction may be due to the specific physiological profile of our BEAS-2B model. As cells of epithelial origin, they may lack the robust inflammasome machinery found in myeloid cells, potentially constraining the cytokine profile within our sampling window. Future studies evaluating post-injury therapeutic regimens, and including both sexes, primary cells and longer observation periods are warranted to fully validate the sustained therapeutic potential of IsoA. Moreover, incorporating primary airway or alveolar epithelial cells, co-culture systems, organoid models, and pathway-specific interventions *in vivo* would help further elucidate the mechanisms underlying the protective effects of IsoA.

## Conclusion

The results of this study demonstrated that IsoA, a natural compound derived from *Inula helenium*, attenuated LPS-induced ALI in mice by reducing inflammatory-cell infiltration and pro-inflammatory cytokines. Moreover, IsoA treatment attenuates MAPK modules and the IL-6/STAT3 axis and mitigates EMT-associated changes in lung tissue. To our knowledge, these data provide the first evidence that IsoA modulates EMT-associated markers in the context of ALI, extending prior research focusing on Nrf2 and TRAF6. Taken together, our findings suggest that IsoA may be a promising candidate for signaling-directed interventions in ALI, and that translational studies in ARDS-relevant models are warranted.

## Supplementary Material

Supplementary figures.

## Figures and Tables

**Figure 1:**
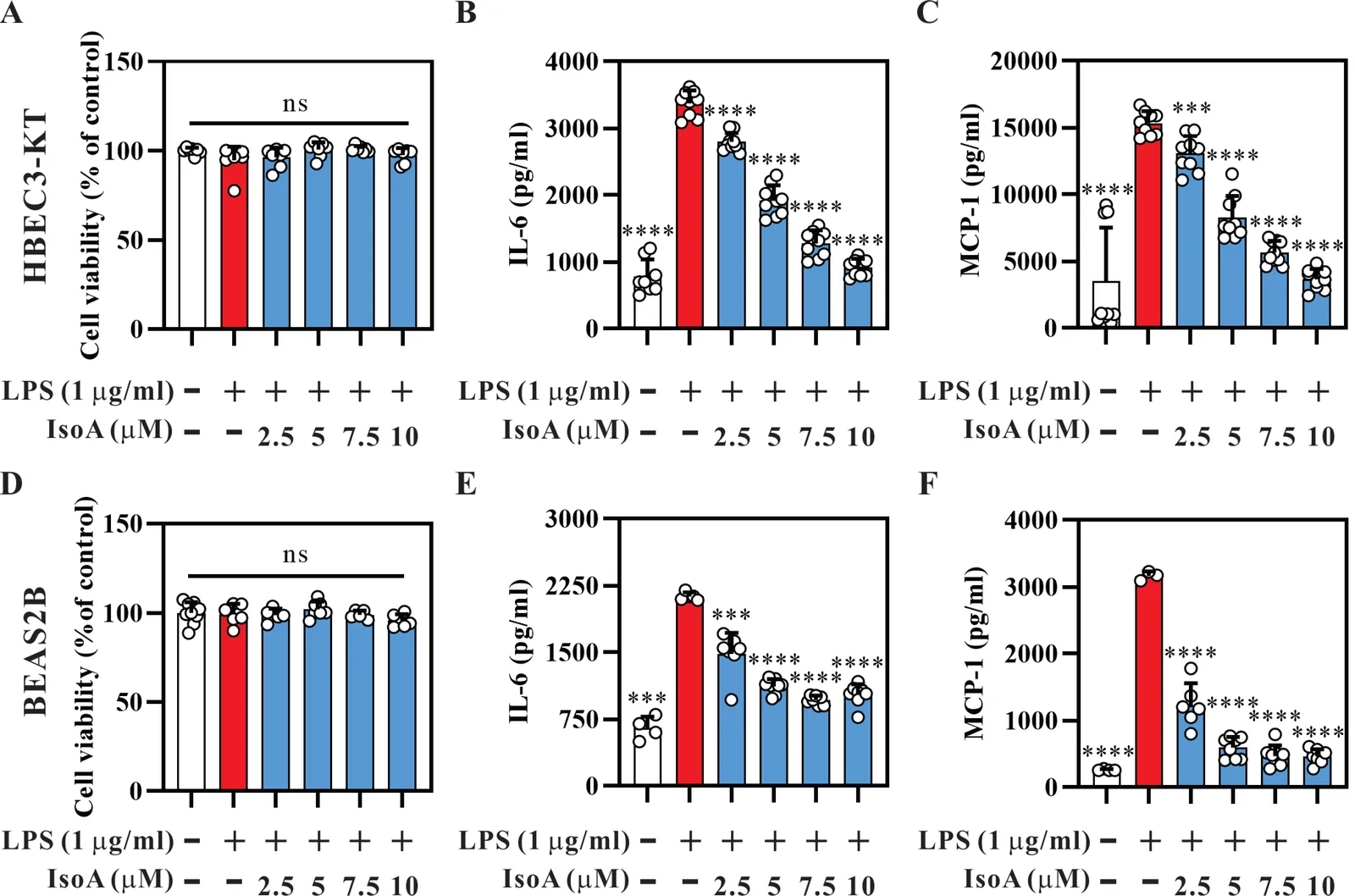
Effects of IsoA on cell viability and pro-inflammatory cytokine secretion of LPS-induced HBEC-3KT and BEAS2B cells. The cell viability of (A) HBEC-3KT and (D) BEAS2B cells was detected by MTT assay. The secretion of IL-6 on LPS-induced (B) HBEC-3KT and (E) BEAS2B cells was determined by ELISA. The production of MCP-1 on LPS-induced (C) HBEC-3KT and (F) BEAS2B cells was determined by ELISA. The values presented are the mean ± SD of six independent experiments, and the significant differences are indicated as * p <0.05, **p <0.01, ***p <0.001, and ****p <0.0001 vs. LPS alone.

**Figure 2:**
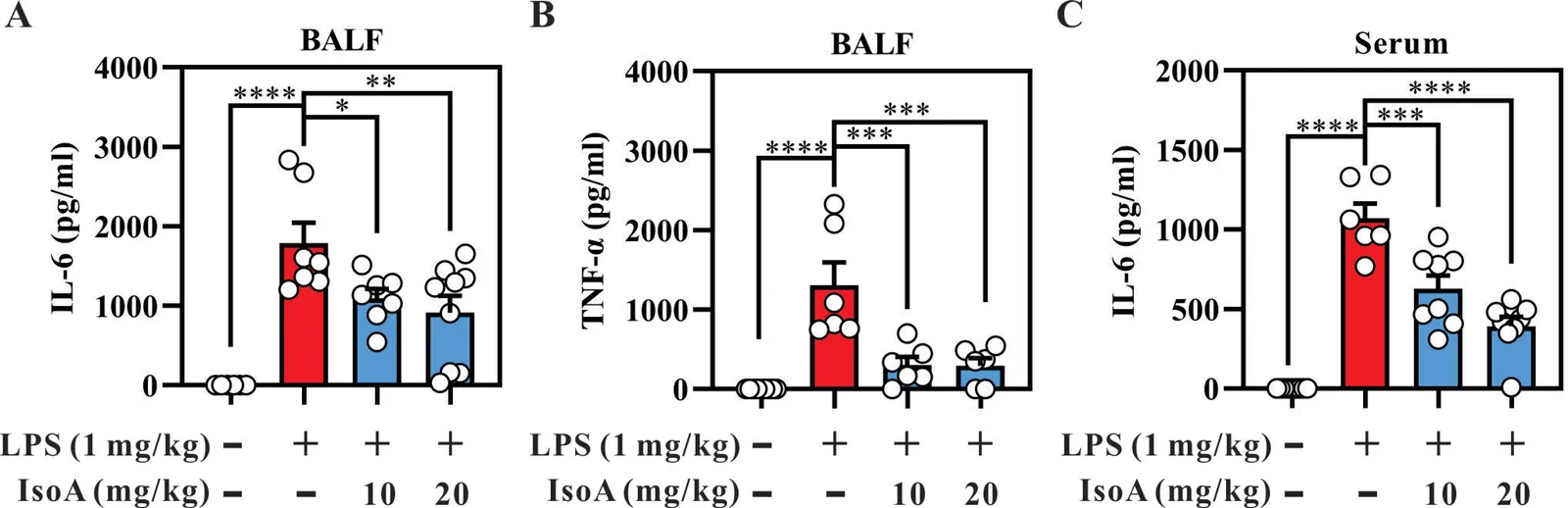
Effects of IsoA on the secretion of pro-inflammatory cytokines in BALF and serum of LPS-induced ALI mice. Mice were intraperitoneally injected with IsoA (10 mg/kg or 20 mg/kg) for 1 h following intratracheal administration of LPS for 4 h. The secretion of (A) IL-6 and (B) TNF-αin BALF and (C) IL-6 in serum of LPS-induced ALI mice was determined by ELISA. The values presented are the mean ± SD of four independent experiments, and the significant differences are indicated as * p <0.05, **p <0.01, ***p <0.001, and ****p <0.0001 vs. LPS alone.

**Figure 3:**
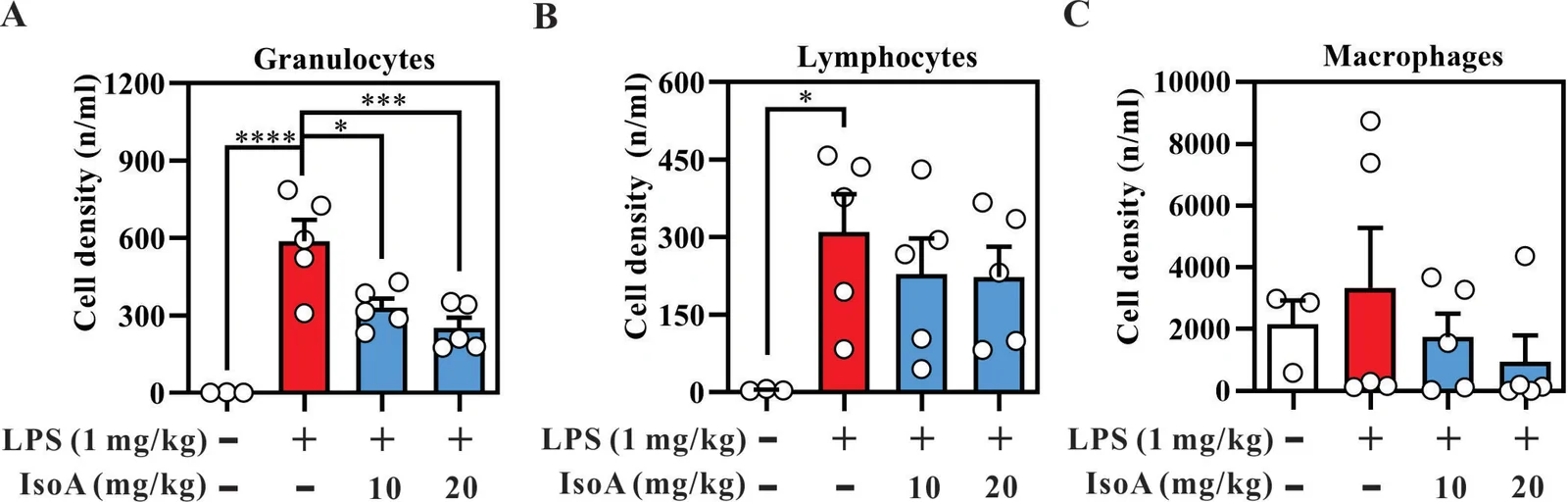
Effects of IsoA on immune cell infiltration in BALF of LPS-induced ALI mice. The cell subsets were identified by multicolor flow cytometry, including the density of (A) granulocytes, (B) lymphocytes, and (C) macrophages. Results are shown as mean ± SD of four independent experiments, and the significant differences are indicated as * p <0.05, **p <0.01, ***p <0.001, and ****p <0.0001, vs. LPS alone.

**Figure 4:**
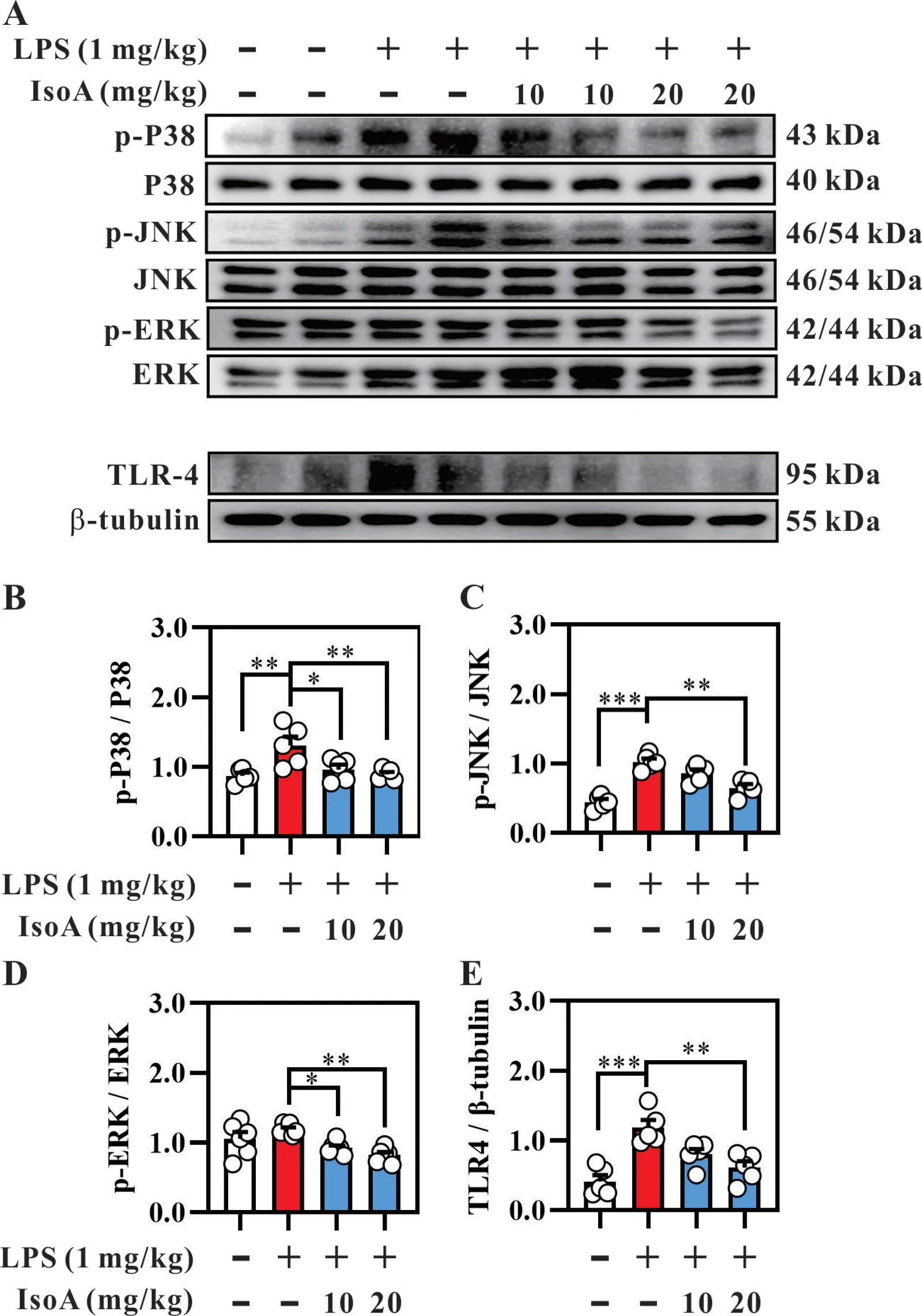
Effects of IsoA on the expression of MAPKs signaling pathway-associated proteins and TLR4 in lung tissue of LPS-induced ALI mice. (A) The expression of MAPKs-related and TLR4 protein. The activation of MAPKs-related protein including (B) p38, (C) JNK, (D) ERK, and (E)TLR4 was examined by Western blot. The values presented are the mean ± SD of four independent experiments, and the significant differences are indicated as * p <0.05, **p <0.01, and ***p <0.001 vs LPS alone.

**Figure 5:**
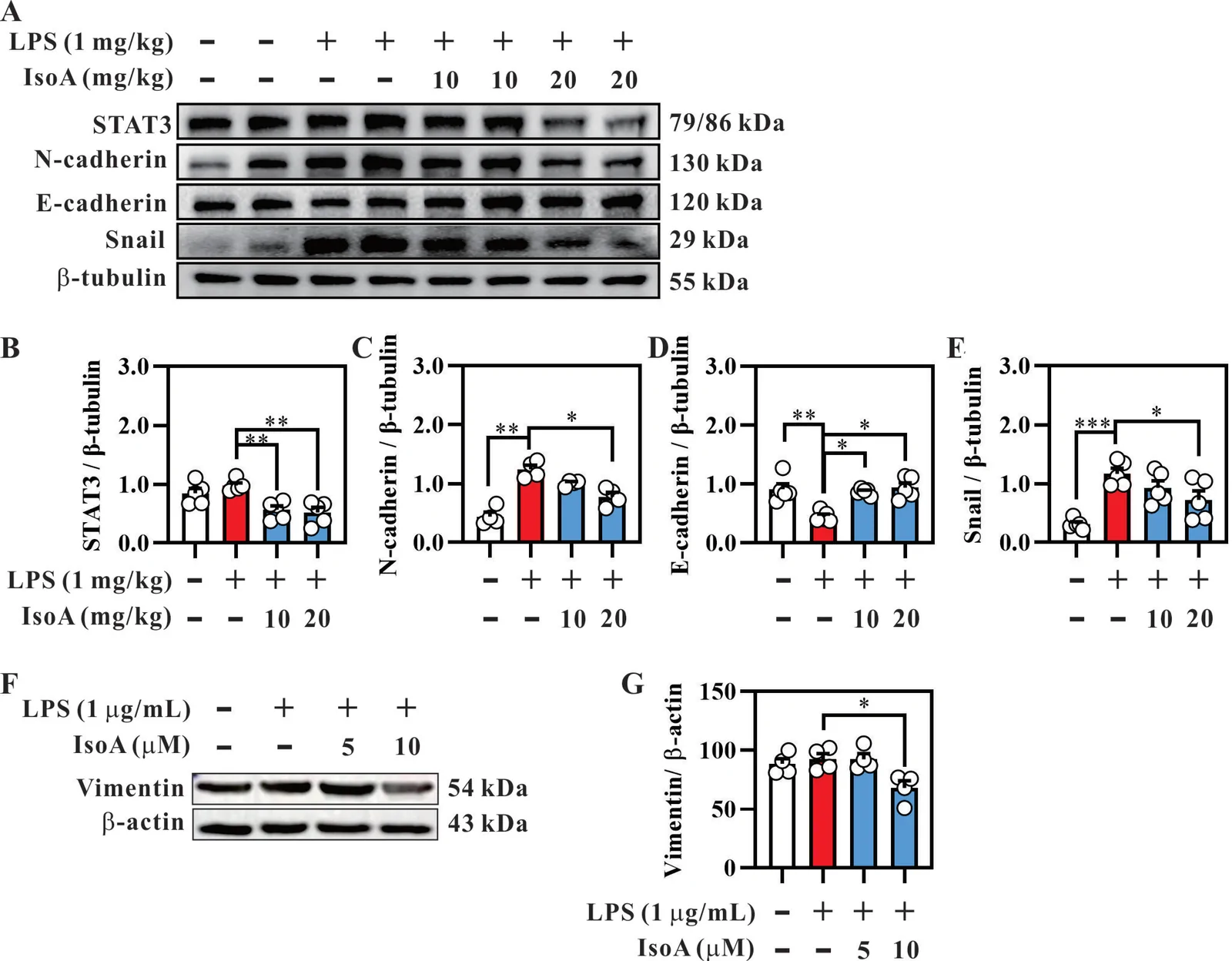
(A) Representative Western blot images showing the protein contents of STAT3, N-cadherin, E-cadherin, and snail in lung tissues 4 h post-LPS instillation. β-tubulin was used as the internal loading control. Molecular weights (kDa) are indicated on the right. (B-E) Densitometric quantification of (B) STAT3, (C) N-cadherin, (D) E-cadherin, and (E) snail protein expression normalized to β-tubulin in lung tissues. (F) Representative Western blot analysis of vimentin expression in BEAS-2B cells pretreated with IsoA (5 or 10 μM) for 1 h followed by LPS (1 μg/mL) stimulation for 24 h. (G) Densitometric quantification of vimentin expression in BEAS-2B cells. Data are presented as mean ± SD. ∗p <0.05, ∗∗p <0.01, and ∗∗∗p <0.001.

**Figure 6:**
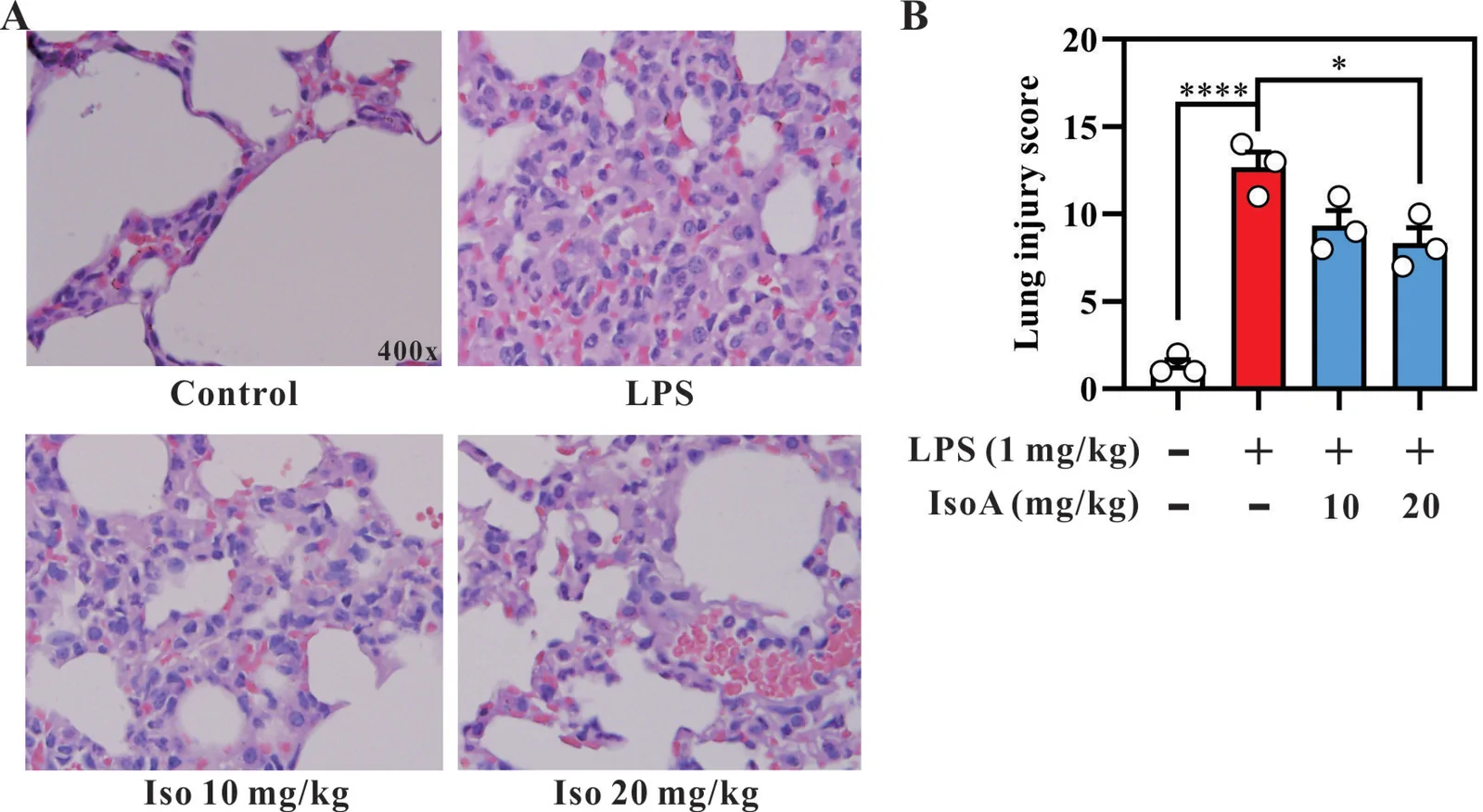
Effects of IsoA on the lung tissue pathology in LPS-induced ALI mice. (A) Representative photographs of the lung tissues stained with HE. Upper left, control, the representative image PBS control. Upper right, the representative image of LPS administration. Lower left, the representative image of low-dose IsoA (10 mg/kg) pretreatment following LPS administration. Lower right, the representative image of high-dose IsoA (20 mg/kg) pretreatment following LPS administration. (B) Morphological changes in lung sections were semi-quantified using lung injury score. The results showed a significant reduction in the severity of lung injury in mice treated with IsoA compared to the LPS-induced ALI mice. The magnification is 400X. The data are shown as mean ± SEM. ***p <0.001 and **** p <0.0001 vs. LPS alone.

## Data Availability

The datasets generated and/or analyzed during the current study are available from the corresponding author on reasonable request.

## Abbreviations

ALI: Acute lung injury
ARDS: Acute respiratory distress syndrome
BALF: Bronchoalveolar lavage fluid
ELISA: Enzyme-linked immunosorbent assay
EMT: Epithelial-mesenchymal transition
ERK: Extracellular signal-regulated kinases
H&E: Hematoxylin and eosin
IL-6: Interleukin-6
HBECs: Human bronchial epithelial cells
JNK: c-Jun N-terminal kinases
LPS: Lipopolysaccharide
IsoA: Isoalantolactone
MAPK: Mitogen-activated protein kinase
MCP-1: Monocyte chemoattractant protein-1
MTT: 3-(4,5-dimethylthiazol-2-yl)-2,5-diphenyl tetrazolium bromide
NF-κB: Nuclear factor kappa-light-chain-enhancer of activated B cells
Nrf2: Nuclear factor erythroid 2-related factor 2
PBS: Phosphate-buffered saline
STAT3: Signal transducer and activator of transcription 3
TNF-α: Tumor necrosis factor-alpha
