# Supplementary material for: Isoalantolactone Attenuates LPS-Induced Acute Lung Injury, an Effect Associated with Suppressed MAPK/STAT3 Activation and Epithelial-Mesenchymal Transition
Source: Int J Med Sci. 2026 Aug 12;23(10):3079–89. doi: 10.7150/ijms.126267 (PMC13617506; doi:10.7150/ijms.126267)
Supplement: Supplementary file 1 [file ijmsv23p3079s1.pdf]

Figure S1. Effects of isoalantolactone (IsoA) on LPS-induced IL-12 and IL-1β release in human bronchial epithelial cells.

Cells were treated with IsoA (5 or 10 μM) in the presence or absence of LPS (1 μg/mL). The concentrations of IL-12 and IL-1β in the culture supernatants were measured. Data are presented as mean ± SEM (n=4). ns, not significant.

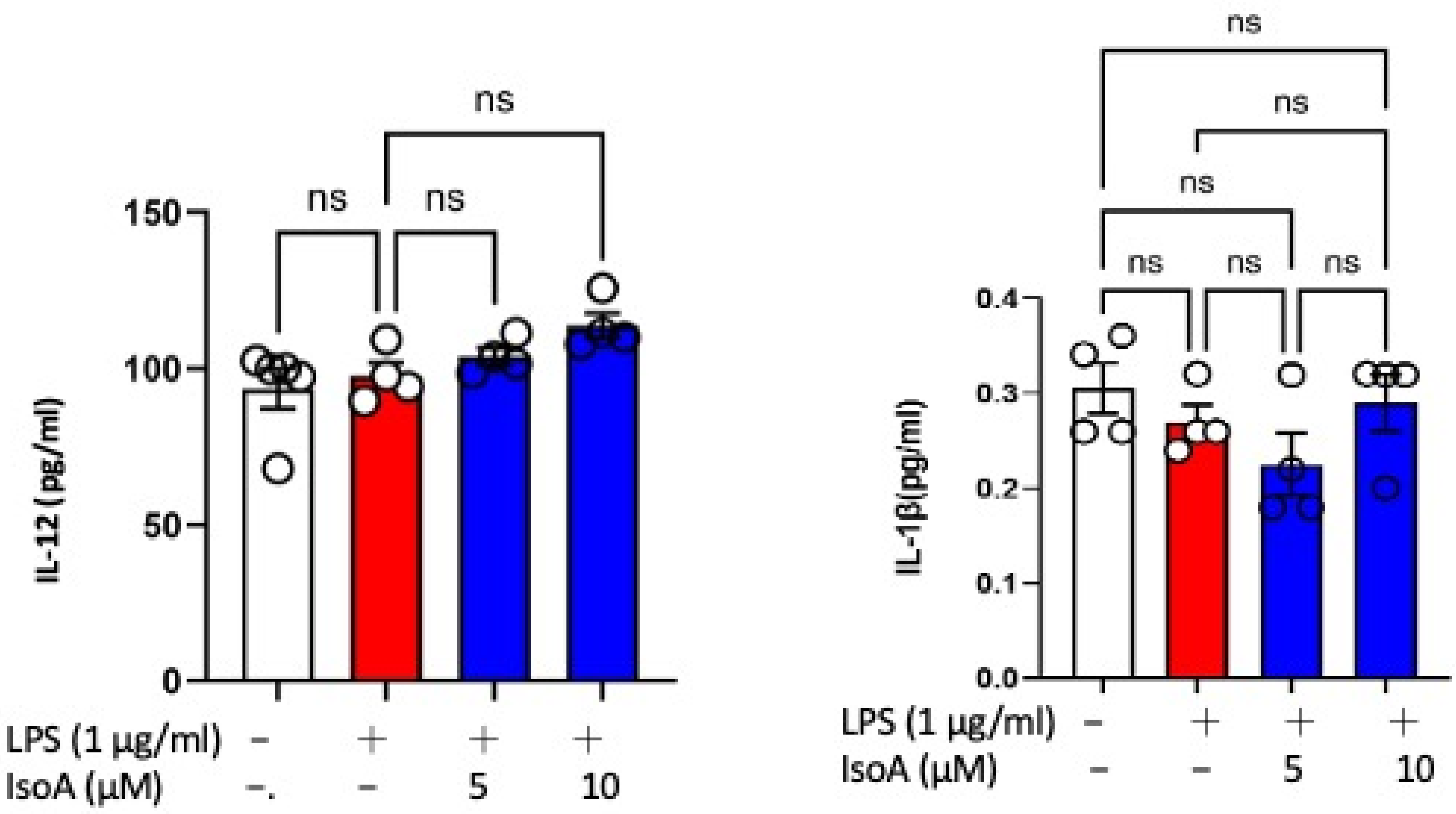
ns
ns
ns
150
100
50
0
IL-12 (pg/ml)
LPS (1 µg/ml) - + + +
IsoA (µM) -. - 5 10
ns
ns
ns
ns
ns
ns
0.4
0.3
0.2
0.1
0.0
IL-1β(pg/ml)
LPS (1 µg/ml) - + + +
IsoA (µM) - - 5 10

Supplementary Figure S2

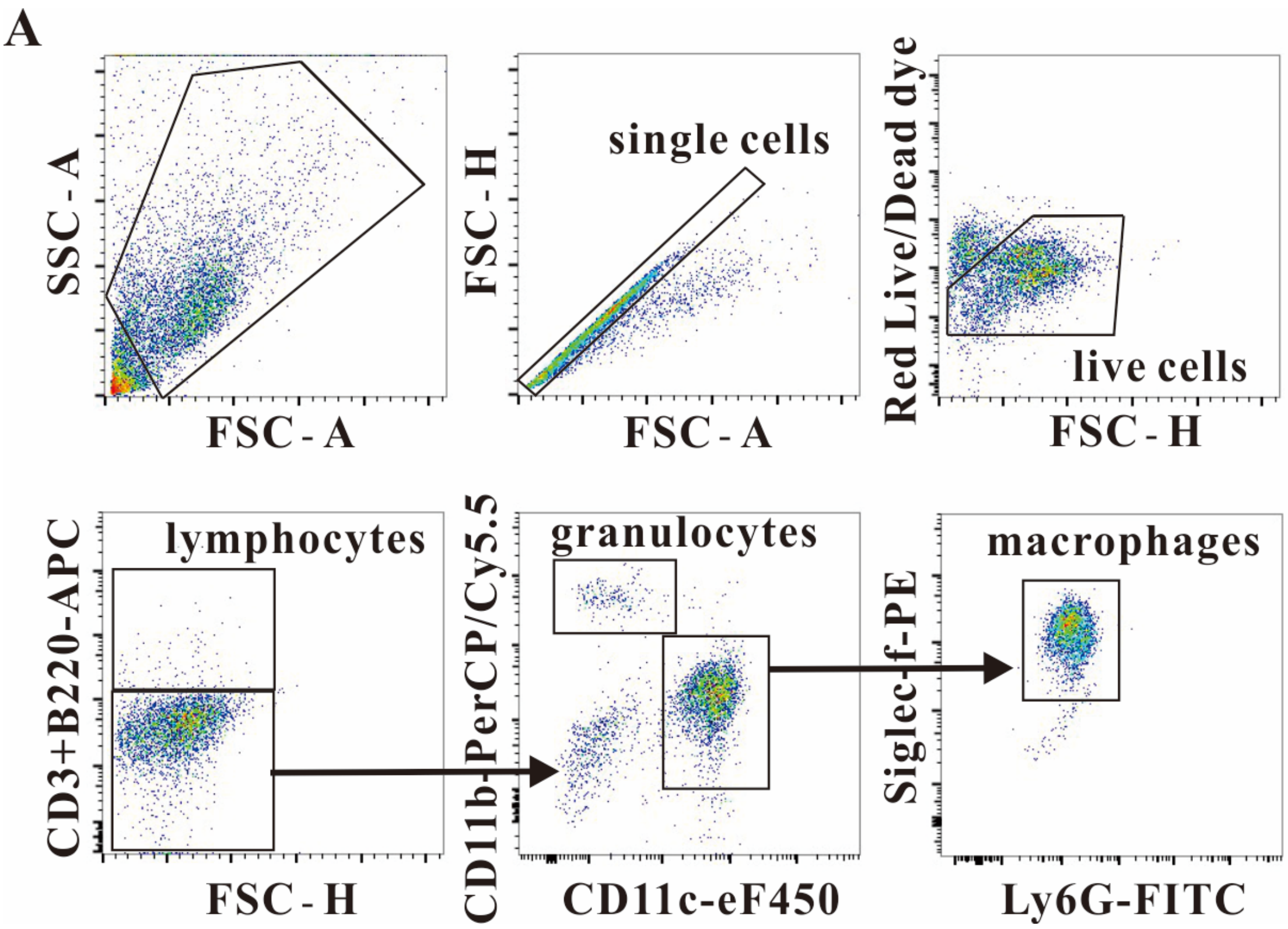

Supplementary Figure 2. Gating strategy for immune cell subsets in BALF. Cells collected from bronchoalveolar lavage fluid (BALF) were first gated based on forward scatter (FSC-A) and side scatter (SSC-A) parameters. Live single cells were then selected for further analysis. Lymphocytes were identified as $CD3^{+}$ or $B220^{+}$ cells. Granulocytes were gated as $CD11b^{+}CD11c^{-}$ cells. $CD11b^{-}CD11c^{+}Siglec\text{-}F^{+}Ly6G^{-}$ cells were defined as macrophages.

Flow cytometry gating strategies

The staining panel and gating strategy used to identify immune cell subsets in BALF were established according to previously published studies [1]. Briefly, BALF cells were incubated with fluorochrome-conjugated monoclonal antibodies at 4 °C for 30 min. The antibodies included FITC-conjugated anti-Ly6G (1A8; BD Biosciences, San Jose, CA, USA), PE-conjugated anti-Siglec-F (E50-2440; BD Biosciences), APC-conjugated anti-B220 (RA3-6B2; BD Biosciences), APC-conjugated anti-CD3 (145-2C11; BD Biosciences), PerCP/Cy5.5-conjugated anti-CD11b (M1/70; BioLegend, San Diego, CA, USA), and eFluor 450-conjugated anti-CD11c (N41B; Invitrogen, Eugene, OR, USA). Cell viability was assessed using Live/Dead Fixable Red dye (Invitrogen). After staining and washing, samples were analyzed by multiparameter flow cytometry using an LSR II instrument (BD Biosciences), and the data were analyzed with FlowJo software (version 10; Tree Star, Ashland, OR, USA). The gating strategy used to identify immune cell populations in BALF is shown in Supplementary Figure 1.

1. Tseng, H.H., et al., *Di-(2-ethylhexyl) Phthalate Promotes Allergic Lung Inflammation by Modulating CD8alpha(+) Dendritic Cell Differentiation via Metabolite MEHP-PPARgamma Axis.* Front Immunol, 2022. **13**: p. 581854.

Figure 4. original western blot for five repeats (MAPK pathway)

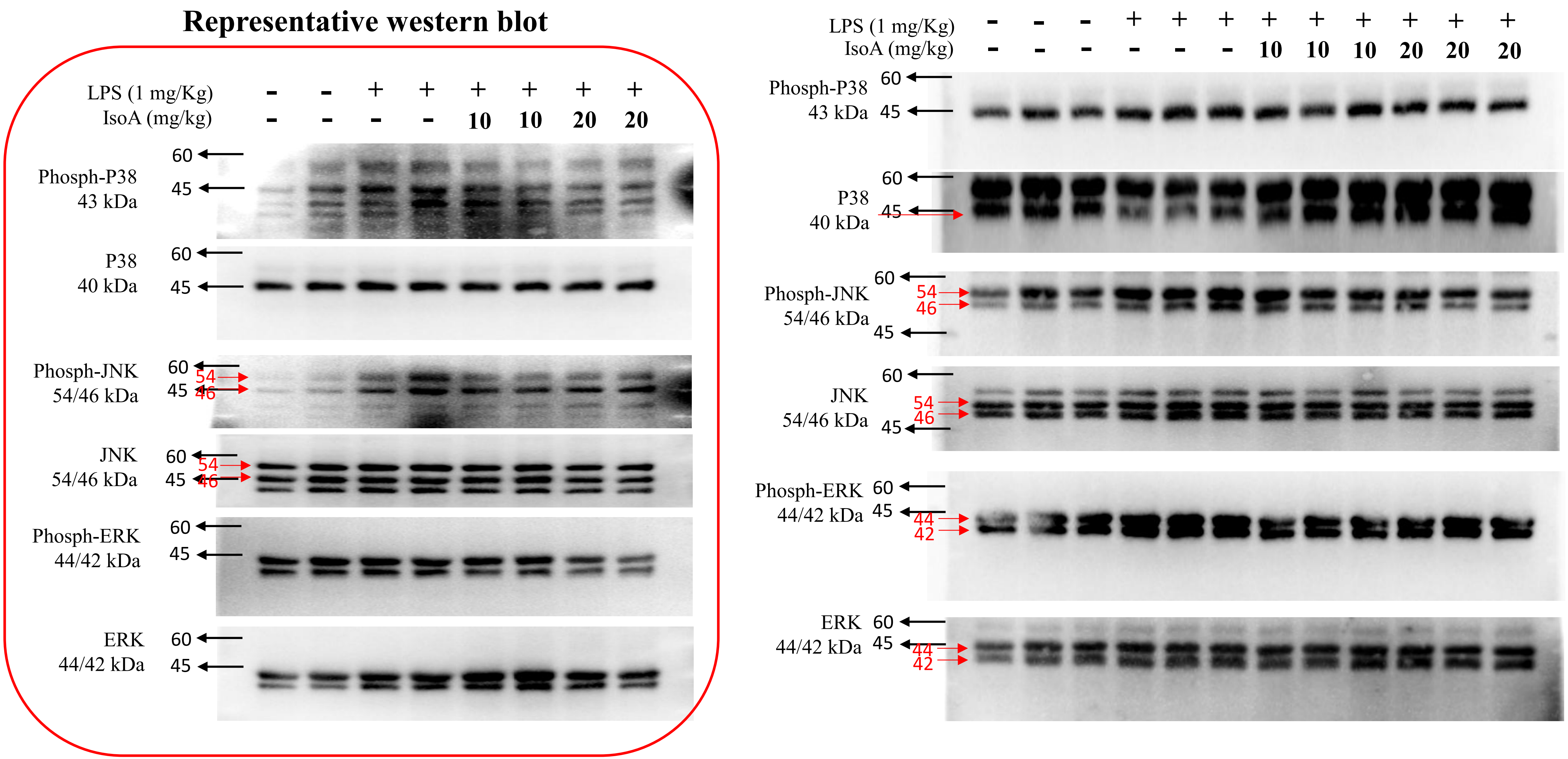

Figure 4. original western blot for five repeats (TLR-4)

Representative western blot

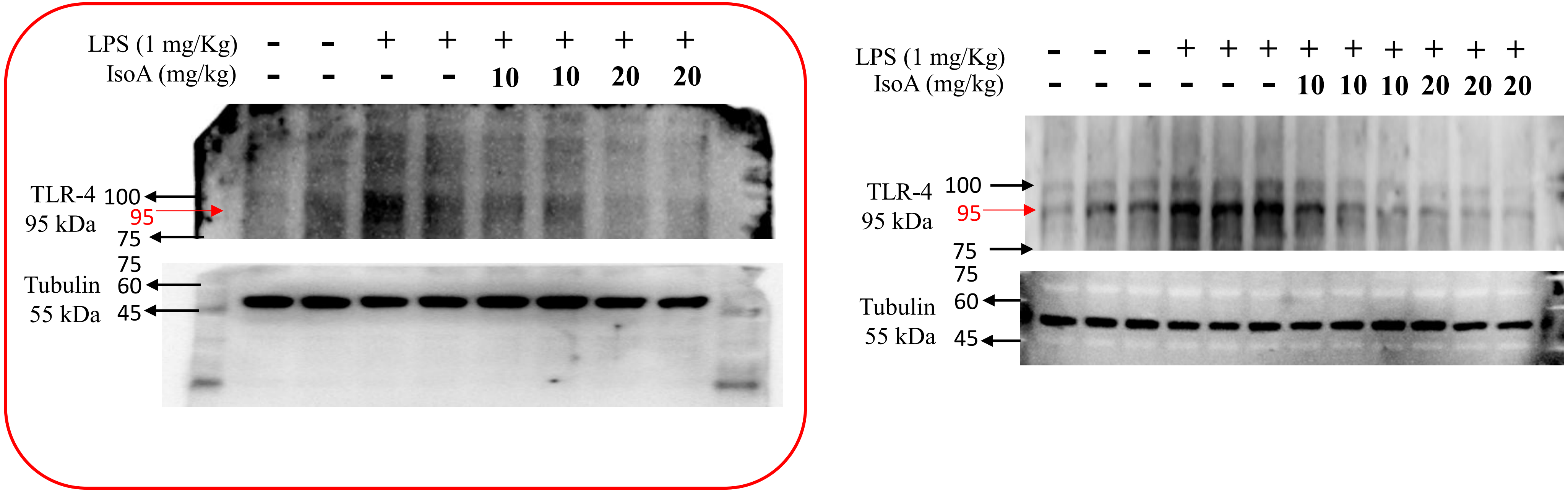

Figure 5. original western blot for five repeats

Representative western blot

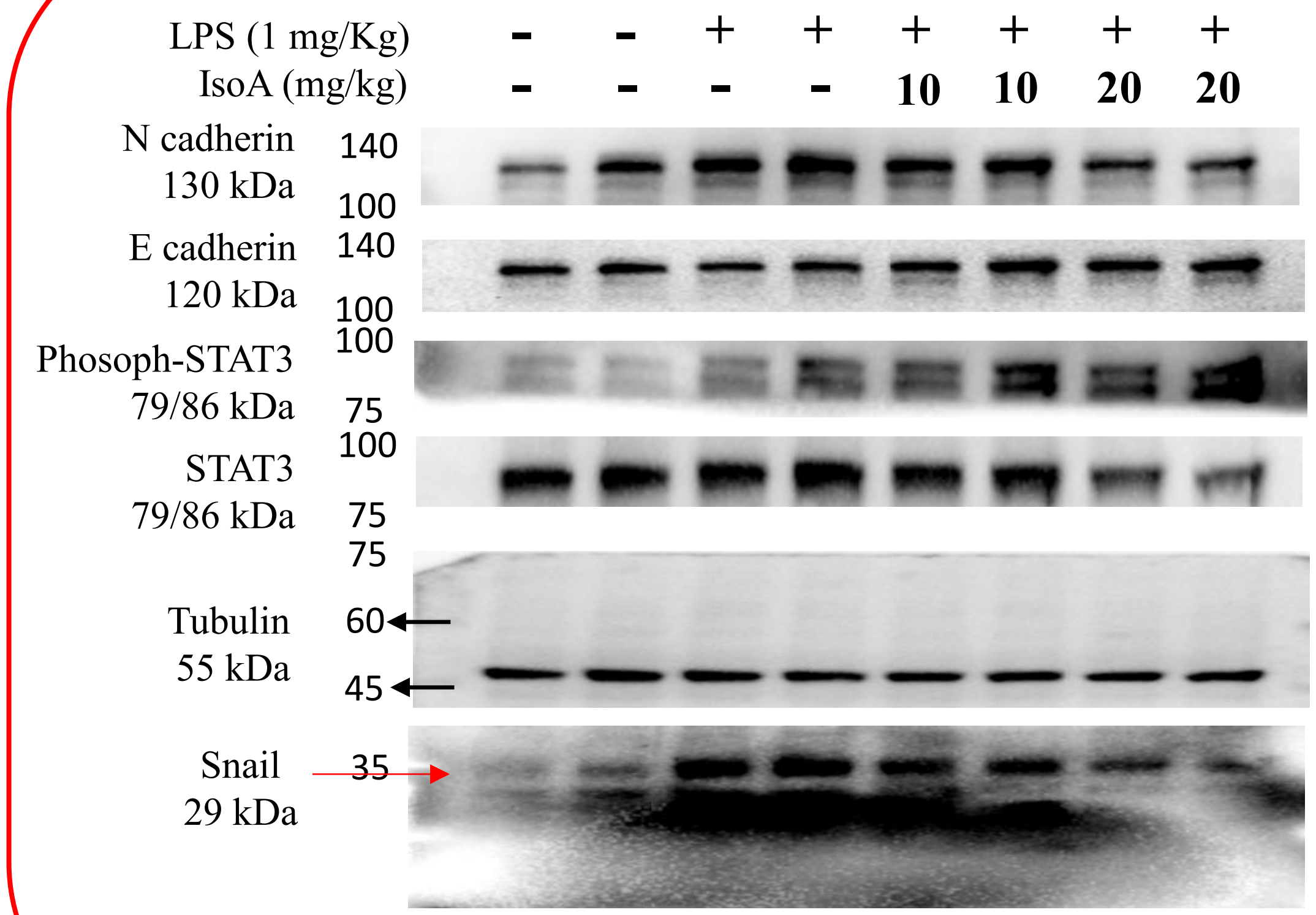

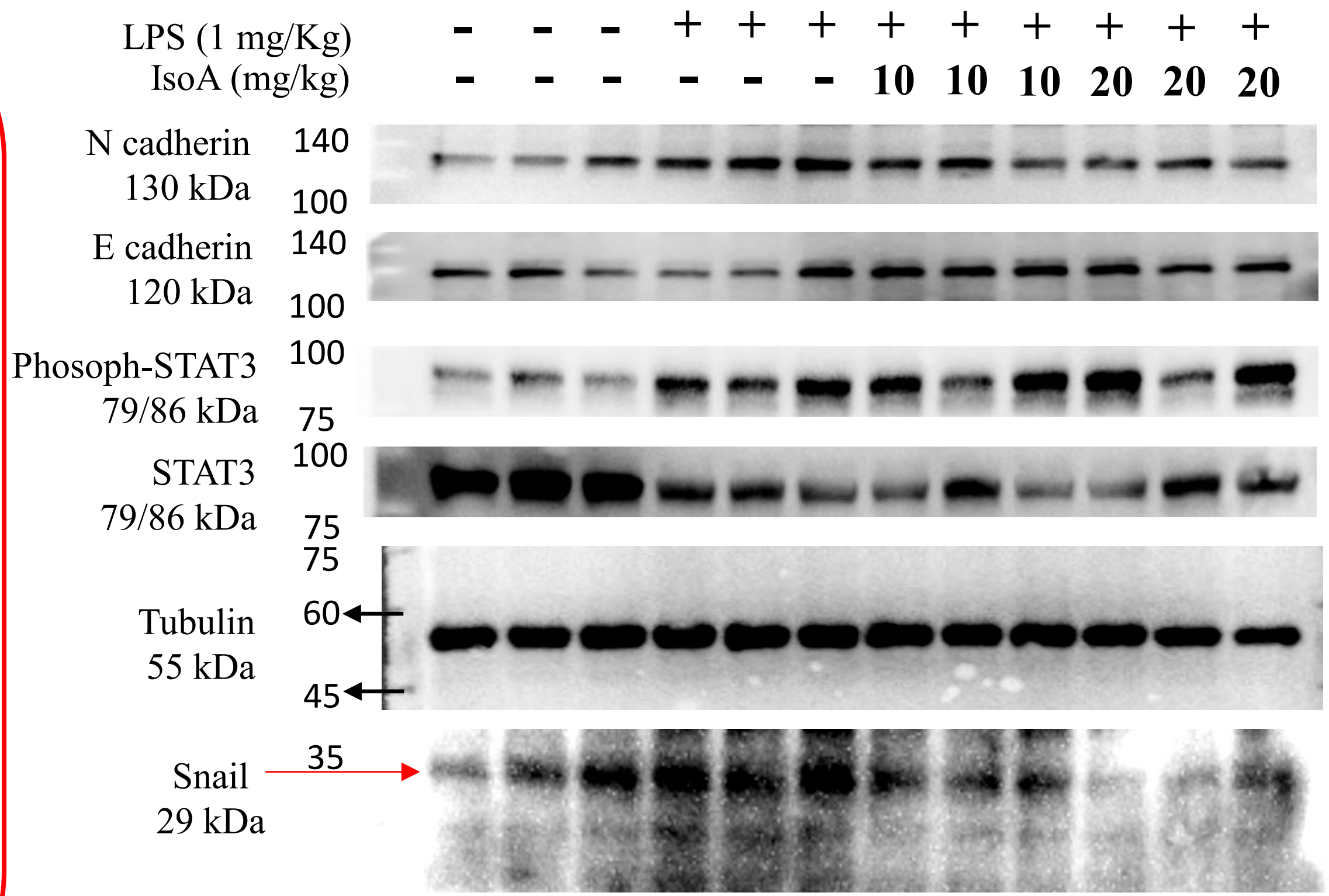

Vimentin expression (raw data)

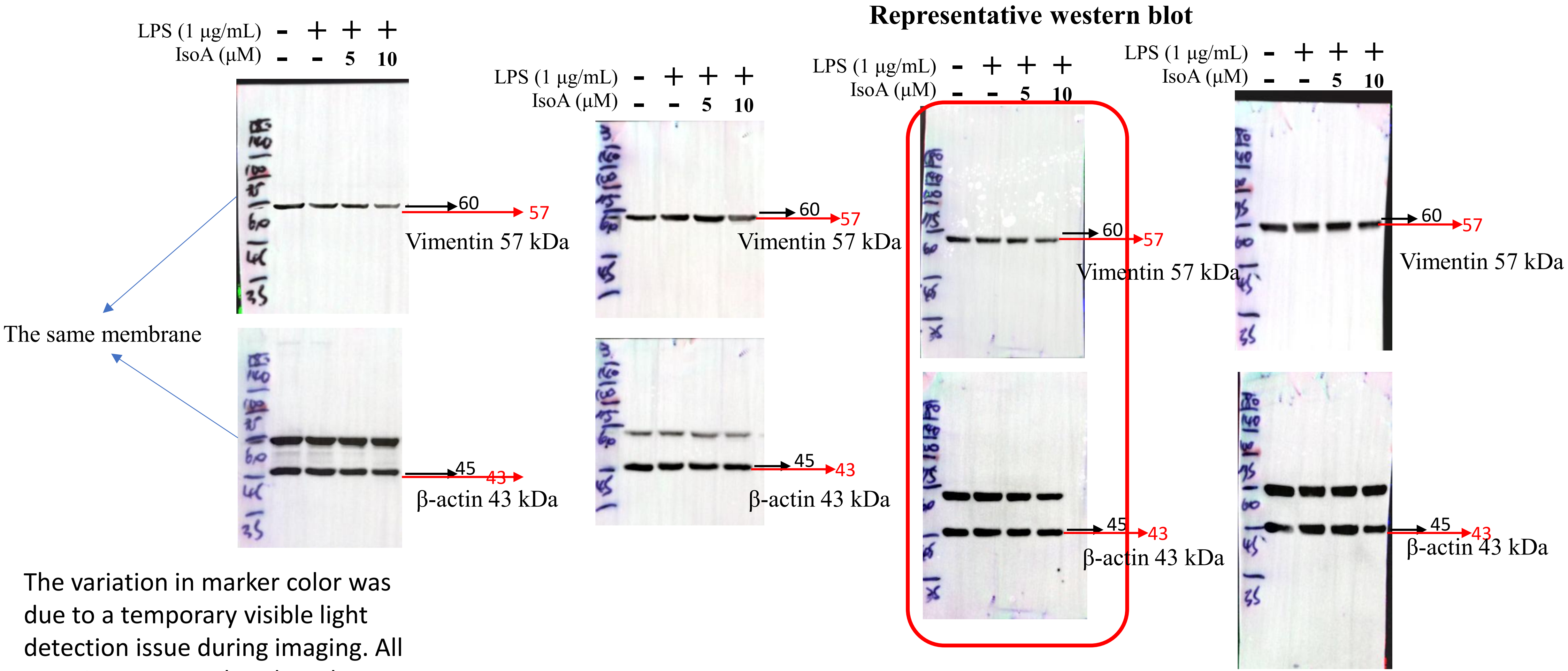

The variation in marker color was due to a temporary visible light detection issue during imaging. All proteins were analyzed on the same membrane.
